# Sodium butyrate in both prevention and supportive treatment of colorectal cancer

**DOI:** 10.3389/fcimb.2022.1023806

**Published:** 2022-10-26

**Authors:** Karolina Kaźmierczak-Siedlecka, Luigi Marano, Elvira Merola, Franco Roviello, Karol Połom

**Affiliations:** ^1^ Department of Surgical Oncology, Medical University of Gdansk, Gdansk, Poland; ^2^ Department of Surgical Oncology, University of Siena, Siena, Italy

**Keywords:** colorectal cancer, short-chain fatty acids, butyrate, sodium butyrate, gut microbiome, metabolome

## Abstract

Accumulating evidence suggests that selected microbiota-derived metabolites play a significant role in both tumor prevention and supportive treatment of cancer. Short-chain fatty acids (SCFAs), i.e., mainly acetate, proprionate, and butyrate, are one of them. Nowadays, it is known that butyrate is a key microbial metabolite. Therefore, in the current review, we focused on butyrate and sodium butyrate (NaB) in the context of colorectal cancer. Notably, butyrate is characterized by a wide range of beneficial properties/activities. Among others, it influences the function of the immune system, maintains intestinal barrier integrity, positively affects the efficiency of anti-cancer treatment, and may reduce the risk of mucositis induced by chemotherapy. Taking into consideration these facts, we analyzed NaB (which is a salt of butyric acid) and its impact on gut microbiota as well as anti-tumor activity by describing molecular mechanisms. Overall, NaB is available as, for instance, food with special medical purposes (depending on the country’s regulation), and its administration seems to be a promising option for colorectal cancer patients.

## Introduction

Short-chain fatty acids (SCFAs) are known as significant microbial metabolites (Mohseni et al., 2020; Fang et al., 2021; Zhang et al., 2022). The group of SCFAs mainly includes acetate (C2), proprionate (C3), and butyrate (C4) (Liu et al., 2018; Guan et al., 2021). They are produced in different amounts; i.e., in the colonic lumen, the proportion is as follows: 60% acetate, 25% proprionate, and 15% butyrate (Canani et al., 2011). An appropriate ratio is 3:1:1 for acetate, proprionate, and butyrate, respectively (Nogal et al., 2021). The highest concentration of SCFAs is observed in the proximal colon (7–140 mM) (Sun et al., 2017; Liu et al., 2018).

Butyrate (a key bacterial metabolite) is produced in the colon through bacterial fermentation using dietary fibers and starch as sources (Louis and Flint, 2017). Butyrate-producing microbes are bacteria belonging to the Firmicutes phylum. According to some data, the most important butyrate-producer is *Faecalibacterium prausnitzii* (Kaźmierczak-Siedlecka et al., 2022). Butyrate provides multiple effects in the human body. Among others, it has anti-inflammatory properties (inhibits the pro-inflammatory mediators TNF-α, IL-1β, IL-6, and IL-8 as well as upregulates the anti-inflammatory IL-10) and affects intestinal mucosal immunity (for instance, through regulation of immune cell migration) (Liu et al., 2018). Sodium butyrate (NaB), which is a salt of butyric acid, is characterized by a wide range of beneficial properties/activities. NaB has been investigated in case of several diseases, such as inflammatory bowel diseases (IBDs) (Chen et al., 2018; Facchin et al., 2020), non-alcoholic fatty liver disease (NAFLD) (Zhou et al., 2017; Zhang et al., 2021), and obesity (Fang et al., 2019; Beisner et al., 2021). During the last several years, an increasing interest in the effect of NaB usage in the context of colorectal cancer has been observed.

Currently, colorectal cancer is one of the most commonly diagnosed cancer. Notably, 1.8 million new cases were noted in 2018 and 1.93 million in 2020, globally (Picard et al., 2020; Li et al., 2022). Despite the fact that the treatment of this cancer is quite well established and it regards neoadjuvant therapy as well as surgery, there is a need to search for new therapeutic mini-invasive or non-invasive methods that, among others, support the preparation of patients for surgical treatment, reduce the side effects of anti-cancer therapy, and inhibit the progression of disease. Modification of gut microbiota and metabolome aspects seems to be extremely significant in the case of colorectal cancer. Using of NaB may be a promising strategy for colorectal cancer patients.

## The general properties/activities of butyrate

### Immune system-related aspects

Butyrate is known as one of the most important metabolites produced through bacterial fermentation in the gut. The biosynthesis of butyrate is done in two metabolic pathways. The first of which regards the phosphorylation of butyryl-CoA to butyryl-phosphate and then the transformation to butyrate with enzyme butyrate kinase. The other metabolic pathway regards the transformation of butyryl-CoA to acetate and then to butyric acid (Liu et al., 2018). It has a wide range of beneficial properties including immunomodulatory functions and anti-tumor activity (Wu et al., 2018; Fu et al., 2019; Siddiqui and Cresci, 2021). It affects immune system by, among others, regulating the expression of pro- and anti-inflammatory mediators. Butyrate inhibits the expression of pro-inflammatory IL-1β and IL-6 while promoting the expression of anti-inflammatory cytokine IL-10 (Hui et al., 2019). Additionally, butyrate promotes the anti-tumor immunity of CD8+ T cells (He et al., 2021). The activity, proliferation, and apoptosis of multiple immune cells may also be directly regulated by butyrate (Danne and Sokol, 2021). Zhou et al. have shown that butyrate is produced by *F. prausnitzii*, and it maintains Th17/Treg balance (Zhou et al., 2018). *Clostridium butyricum*, which has probiotic properties, is also known as a butyrate-producing bacterium (Chen et al., 2020; Stoeva et al., 2021). In the Li et al. study, it was observed that butyrate constrained neutrophil functions as well as ameliorated mucosal inflammation in case of inflammatory bowel disease (Li et al., 2021). Butyrate provides anti-inflammatory effects and increases apoptosis in cancer cells (Jaye et al., 2022). Butyrate and proprionate are more effective in inhibiting HT29 cell growth compared to acetate (Jaye et al., 2022). Moreover, butyrate is a main source of energy for colonocytes (Fu et al., 2019). Additionally, it influences mucosal immunity and maintains gut microbiota stability, providing gut microbial homeostasis (Wu et al., 2018; Fu et al., 2019).

### Maintenance of intestinal barrier integrity

Epithelial cells have G-protein-coupled receptors (GPCRs), which are the receptor of butyrate (Hanus et al., 2021). It modulates cellular functions by the above-mentioned GPCRs, which are expressed in multiple cells and tissues (for instance, adipose tissue, monocytes, neutrophils, B and T lymphocytes, and colonic myeloid cells) (Siddiqui and Cresci, 2021). In adipose tissues, butyrate promotes production and secretion of leptin; thus, it may be mentioned as a body weight regulator (Coppola et al., 2021). Butyrate enhances epithelial cell proliferation and the mucus layer, and improves tight junctions (Peng et al., 2009). Notably, tight junctions are intercellular junctions essential for maintaining epithelial barrier integrity (Otani and Furuse, 2020). The claudin family of membrane proteins are a significant part of tight junctions (Otani and Furuse, 2020). The mucus layer is made up of butyrate, mucins, immunoglobulins, and glycoproteins (Zhang et al., 2019). It should be emphasized that butyrate upregulates the expression of the MUC2 gene; thus, it promotes the synthesis of mucins, which protect the epithelial cells from lumen toxins (Zhang et al., 2019). Bacteria such as *Fusobacterium nucleatum*, *Bacteroides fragilis*, and *Escherichia coli* are known colorectal cancer-associated pathogens, and they contribute to the dysfunction of intestinal barrier integrity (Kaźmierczak-Siedlecka et al., 2020; Ahmad Kendong et al., 2021). Butyrate increases the expression of claudin-1 and Zonula Occludens-1; thus, it is important in the context of maintenance of intestinal barrier integrity (Hajjar et al., 2021). In the Feng et al. study (regarding Caco-2 monolayers), it has been revealed that acetate (0.5 mM), proprionate (0.01 mM), and butyrate (0.01mM) both alone or in combination significantly increase transepithelial electrical resistance (Feng et al., 2018). They protect intestinal barrier function by inhibition of NLRP3 (cytosolic protein) inflammasome (Feng et al., 2018; Sharma and Kanneganti, 2021). Nevertheless, according to Huang et al., butyrate is the main SCFA that alleviates intestinal barrier dysfunction by downregulating the level of claudin-2 (Huang et al., 2021). Moreover, the authors concluded that claudin-2 is the major target of butyrate (Huang et al., 2021). The improvement of the intestinal barrier after butyrate supplementation was also confirmed in the Zhao et al. study regarding 48 rats with severe acute pancreatitis and intra-abdominal hypertension (Zhao et al., 2020). In this study, *C. butyricum* (butyrate-producing probiotic bacteria) and butyrate were given orally. It was noted that rats that consumed *C. butyricum* or butyrate had reduced intestinal injury and decreased the plasma level of inflammatory cytokines, diamine oxidase, and lipopolysaccharide (Zhao et al., 2020).

To summarize this part, butyrate is a key microbial metabolite that extremely affects the intestinal barrier function, and it seems to be needed for colorectal cancer patients in which increased intestinal permeability is noted.

### Butyrate and apoptosis of cancer cells

Butyrate can induce the apoptosis of colorectal cancer cells (Bordonaro, 2020). In the Xiao et al. study, the apoptosis of colon cancer cells (HCT116) was detected using flow cytometry (Xiao et al., 2014). These cells were exposed to NaB at a dose of 10 mmol/L per 24 h. Previously, colon cancer cells were also treated with ERK inhibitor or siRNA. It was noted that NaB modulates ERK and sphingosine kinase 2 and consequently induces apoptosis of colon cancer cells (Xiao et al., 2014). The anti-carcinogenic effect of butyrate on colon cancer cells (SW480) was also confirmed in the Elimrani et al. study (Elimrani et al., 2015). Similarly, Roy et al. have shown that butyrate at a dose of 2.5–20 mM (and also carnitine) induces apoptosis of colon cancer cells and inhibits Caco-2 cell proliferation (Roy et al., 2009). Taking together the results of the above-mentioned studies, it can be concluded that butyrate may provide anti-carcinogenic effects.

### Impact on anti-cancer treatment

Recently, it was shown that butyrate may improve the efficiency of radiotherapy (Park et al., 2020). Moreover, butyrate may enhance the irinotecan effect; thus, it may enhance the effect of chemotherapy (Encarnação et al., 2018). Similarly, the results of another study revealed that butyrate promotes the effects of 5-fluorouracil on cancerous colonocytes (Geng et al., 2021). It is estimated that approximately 40% of patients who underwent chemotherapy developed mucositis (Ferreira et al., 2012). Butyrate can reduce the side effects of treatment with 5-fluorouracil, which was shown in a mouse model study with mucositis induced by chemotherapy (Ferreira et al., 2012).

The reduction of butyrate-producing bacteria is observed in colorectal cancer patients (Wang et al., 2012). The intracellular concentration of butyrate in colonic cells is regulated by transporters, such as monocarboxylate transporter 1 (MCT1), sodium-coupled monocarboxylate transporter 1 (SMCT1), and breast cancer resistance protein (BCRP) (Gonçalves and Martel, 2016). Notably, the alterations of these transporters’ expression may occur in colorectal cancer patients (Gonçalves and Martel, 2016). During the discussion of transporters MCT1 and SMCT1, the term “butyrate paradox” was mentioned. The basis of this paradox is found deeply in epigenetics (Salvi and Cowles, 2021). In case of differentiated intestinal epithelial cells, butyrate is oxidized and utilized as a fuel (energy), and in this time, it is not able to inhibit histone deacetylase (HDAC), whereas cancerous colon cells use preferentially glucose as fuel instead of butyrate (Salvi and Cowles, 2021). Then, butyrate accumulates and acts as an inhibitor of HDAC. There is an observed prevalence of glycolytic metabolism over oxidative phosphorylation in these cells. The modification of histone (H3), which is induced by butyrate, is linked to the activation of genes participating in both cell cycle inhibition and apoptosis (Salvi and Cowles, 2021).

**Figure 1:**
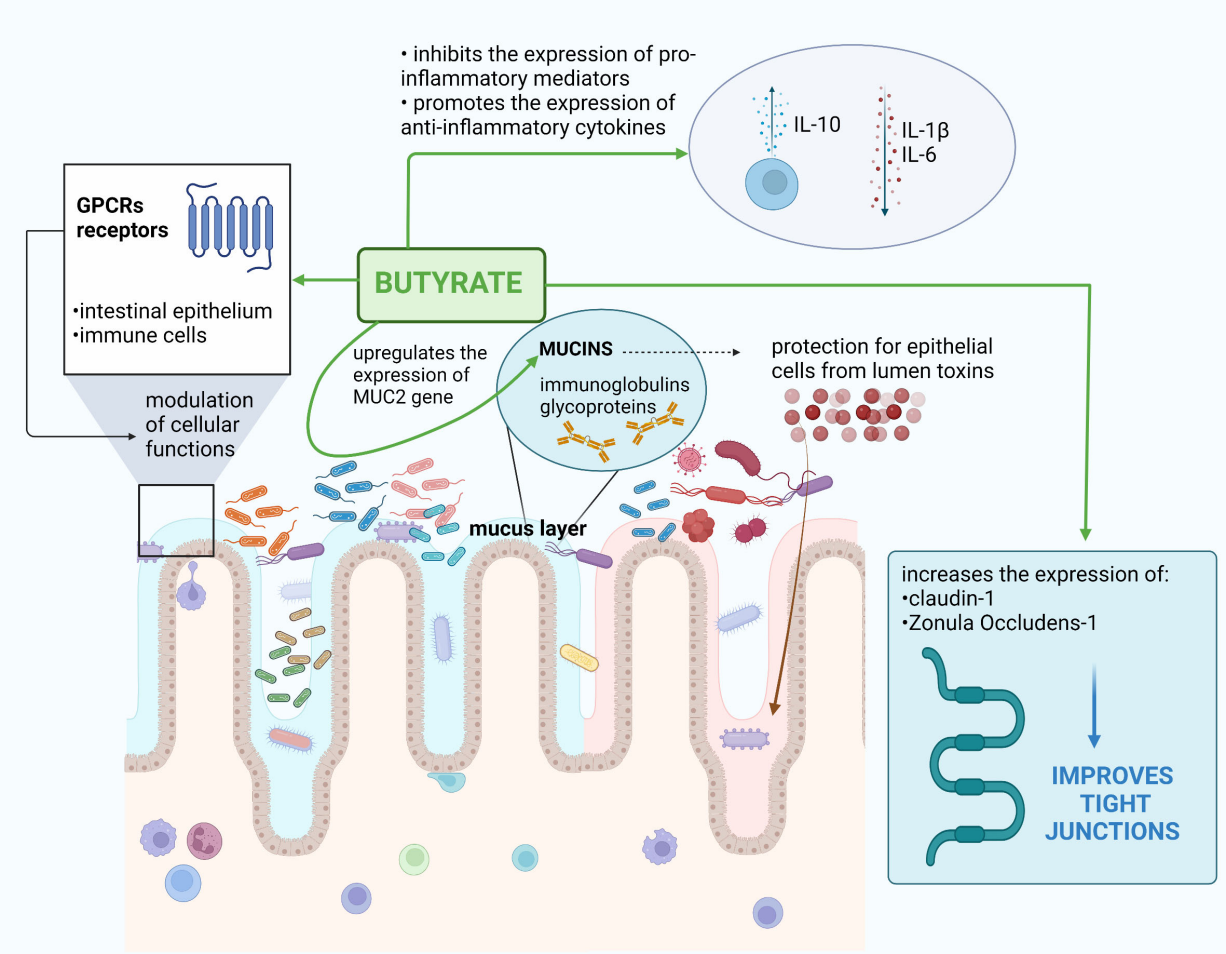
Butyrate-associated beneficial effects in oncological aspects. Own elaboration based on literature (Gonçalves and Martel, 2016; Fu et al., 2019; Hui et al., 2019; Zhang et al., 2019; Hajjar et al., 2021; Huang et al., 2021; Siddiqui and Cresci, 2021).

The summary of butyrate properties is presented in **Figure 1**.

## Sodium butyrate

### Anti-tumor activity of NaB

NaB is able to provide an anti-tumor effect in the context of colorectal cancer. Notably, there are several mechanisms by which NaB may be involved in this process (Wang et al., 2020). In the Xi et al. study, the effect of NaB on colorectal cancer cells (lines SW480, LOVO, HCT116, and HCT8) was investigated (Xi et al., 2021). The results of this study explore the anti-cancer activity of this component; i.e., NaB is able to induce apoptosis as well as inhibit colorectal cancer cell proliferation (Xi et al., 2021). In another study, the impact of NaB on colorectal cell lines (HCT116 and SW480) was also assessed (Zhou et al., 2019). The authors used next-generation RNA sequencing. It was noted that 7,192 genes were differently expressed in cells treated with NaB in comparison with untreated cells. Recently, it was also observed that NaB is able to inhibit colorectal cancer cells’ (lines HCT116 and LOVO) migration through enhancement of miR-200c expression-mediated downregulation of Bmi-1 (Xu et al., 2018). Notably, in another study it was noted that the miR-200c/FUT4 axis prevents the proliferation of colon cancer cells (Cong et al., 2021). This effect is obtained by downregulation of the Wnt/β-catenin pathway (Cong et al., 2021). Interestingly, Sanaei et al. investigated the effect of NaB on both AsPC-1 (pancreatic cancer) and HCT-116 (colon cancer) cell lines (Sanaei and Kavoosi, 2022). It was observed that NaB increased p16INK4a, p14ARF, and p15INK4b, and reduced class I and II HDACs (Sanaei and Kavoosi, 2022). The authors also reported that colon cancer cell lines were more sensitive to NaB than pancreatic cell lines (Sanaei and Kavoosi, 2022). Notably, p16INK4a is a tumor suppressor protein (Serrano, 1997). It was observed that the loss of p14ARF expression is related to more advanced tumors, which has been confirmed in the Kim et al. study (Kim et al., 2020) (**Table 1**).

**Table 1:** Summary of the anti-tumor activity of NaB. CRC—colorectal cancer.

Cells lines	NaB’s mechanism of action	Reference
CRC cells lines: SW480, LOVO, HCT116, HCT8	➢ induces apoptosis ➢ inhibits CRC cell proliferation	Xi et al., 2021
CRC cells lines: HCT116, SW480	➢ 7,192 genes were differently expressed in cells treated with NaB in comparison with untreated cells	Zhou et al., 2019
CRC cells lines: HCT116, LOVO	➢ inhibits CRC cell migration *via* enhancement of miR-200c expression-mediated downregulation of Bmi-1	Xu et al., 2018
Pancreatic cell line: AsPC-1, colon cell line: HCT-116	➢ increases p16INK4a, p14ARF, and p15INK4b	Sanaei and Kavoosi, 2022
CRC cell lines: HT29, SW480	➢ NaB significantly inhibits the growth of CRC cells and decreases the expression of thioredoxin-1 (Trx-1) protein in these cells	Wang et al., 2020
CRC cell lines: HCT-116, HT-29	➢ induces autophagy in CRC cells by LKB1/AMPK signaling	Luo et al., 2019

Thioredoxin and thioredoxin reductase are important modulators of tumor development (Zhang et al., 2017). Wang et al. have reported that NaB significantly inhibited the growth of colorectal cancer cells as well as decreased the expression of thioredoxin-1 (Trx-1) protein in these cells (Wang et al., 2020). Notably, these results were not observed in normal colon epithelial cells and that can explain the selective inhibition of cell growth by NaB (Wang et al., 2020). Trx-1 is defined as a small redox-active protein (Shao et al., 2020; Liu et al., 2022). The downregulation of Trx-1 seems to be important in the context of colorectal cancer (Wang et al., 2020). It can be associated with inflammation and oxidative stress (Shao et al., 2020). According to the results obtained in the Shao et al. study, Trx-1 can also be a potential therapeutic target in case of sepsis, due to the fact that it plays a significant role in inflammation and oxidative stress (Shao et al., 2020).

The deficiency of folic acid alters the cytosine methylation in DNA (Lu et al., 2008). Recently, Lu et al. investigated the role of folic acid and NaB in the prevention of colorectal cancer in a mouse model study (Lu et al., 2008). The results of this study have shown that the lower level of p21WAF1 gene expression was found in colorectal cancer samples compared to normal colorectal mucosa. The administration of NaB beneficially increased the level of p21WAF1 mRNA and p21WAF1 protein. Thus, it can prevent tumorigenesis in a mouse model of colorectal cancer induced by 1,2-dimethylhydrazine (Lu et al., 2008).

AMPK regulates glucose and cholesterol metabolism (Shackelford and Shaw, 2009). Liver kinase B1 (LKB1) is a tumor suppressor gene on human chromosome 19p13. LKB1 controls both cell metabolism and oxidative stress, and together with AMPK, it controls cell growth (Shackelford and Shaw, 2009; Ciccarese et al., 2019). NaB induces autophagy in colorectal cancer cells by LKB1/AMPK signaling, which has been demonstrated in the Luo et al. study (Luo et al., 2019). The authors concluded that NaB may be a novel target for colorectal cancer patients (Luo et al., 2019).

### NaB and its impact on gut microbiota

The supplementation with NaB can beneficially affect gut microbiota. In the Ma et al. study, the effect of NaB on modulation of gut microbiota in mice with colorectal cancer liver metastasis was investigated (Ma et al., 2020). The composition of gut microbiota was assessed using 16S rRNA gene sequencing. It was observed that NaB beneficially altered gut microbiota. Moreover, it modulated immune system by decreasing Treg cells and increasing NK cells as well as T helper cells (Ma et al., 2020). In another animal model (C57BL/6J mice) study, it was revealed that exercise and NaB supplementation reversed metabolic dysfunctions, which have been induced by high-fat diet (*p* < 0.05), and inhibited the amount of microbes producing lipopolysaccharide (*p* = 0.001) (Yu et al., 2019).

## The impact of diet on the concentration of SCFAs

The “Western” diet, which regards the consumption of high amounts of saturated fatty acids, simple sugars, and highly processed food, negatively alters the gut microbiome, causing its imbalance and promoting inflammatory environment (Bibbò et al., 2016; Christ et al., 2019). This type of diet is associated with an increased amount of opportunistic bacteria, lipopolysaccharide, and trimethyloamine-N-oxide as well as the decrease of SCFA production (Beam et al., 2021). As a consequence, it contributes to maintain chronic inflammation and development of nutrition-related diseases. Recently, it was shown that the dysbiotic microbial changes that are caused by a “Western” type of diet can be reversed by supplementation with butyrate (van den Berg et al., 2021). These results were obtained in an animal model study (C57BL/6 mice) (van den Berg et al., 2021).

The amount of bacteria that produce butyrate may be increased by the administration of omega-3 fatty acids [eicosapentaenoic acid (EPA) and docosahexaenoic acid (DHA)] (Gheorghe et al., 2022). In the Zhuang et al. study, it was noted that both EPA and DHA contribute to the increase of butyrate-producing bacteria while decreasing the bacteria that produce lipopolysaccharide, such as *Bilophila* and *Escherichia/Shigella* (Zhuang et al., 2020). Overall, omega-3 fatty acids have an impact on the composition of gut microbiota (Costantini et al., 2017). Moreover, they improve intestinal barrier integrity and reduce the amount of bacteria-producing trimethylamine (TMA) (Rousseau, 2021). The Mediterranean diet, which regards the consumption of food with a high content of dietary fiber, fish, olives, and olive oil (thus also omega-3 fatty acids), is associated with a high level of bacteria that produce SCFAs, and as a consequence, it contributes to the production of a high level of SCFAs and provides gut homeostasis (Merra et al., 2020; Calabrese et al., 2021; Gibiino et al., 2021; Nogal et al., 2021).

## Directions for the future and recommendations for clinical practice in the context of microbiota

First of all, specialists should take into consideration the analysis of gut microbiota-related aspects in case of colorectal cancer patients. They should put more attention to fungi and viruses instead of only bacteria, because they are also a significant part of gut microbiota participating in carcinogenesis. According to some data, the components of gut microbiota can be used as novel biomarkers for the early detection of tumors, or they can be a promising marker for anti-cancer treatment including immunotherapy (Temraz et al., 2019; Tsiaoussis and Souglakos, 2021). Moreover, specialists should search for not only a local but also a distal association. For instance, there is a link between the imbalanced changes of oral microbiota and colorectal carcinogenesis and the progression of colorectal cancer. The gut microbial community and key phenotypes are also a significant field that should be further discussed. Notably, microbes can modulate host phenotypes (Han et al., 2021). Consequently, it affects many pathways and even changes the response to immunotherapy (Han et al., 2021). In this context, the production of metabolites by gut microbiota seems to be crucial. Therefore, not only the composition of gut microbiota but also microbiota-derived metabolites should be analyzed in colorectal cancer patients. The specialists should consider the supplementation of NaB in colorectal cancer but in combination with components that stimulate the production of butyrate, such as not only dietary fiber but also omega-3 fatty acids. Notably, these data are still deeply undiscovered. Some of the products/supplements (not only probiotics) can also change gut microbiota and even the microenvironment of the tumor. Interestingly, oral immunonutrition can alter the microenvironment of the tumor, which has been shown in the D’Ignazio et al. study (D’Ignazio et al., 2020).

Another challenge for the future of gut microbiota is shallow shotgun sequencing. Currently, in most of the studies, gut microbiome is analyzed with 16S rRNA gene sequencing, whereas the shallow shotgun method allows one to obtain a more functional point of view.

## Conclusions

SCFAs are an integral part of gut microbiome functioning/homeostasis. Butyrate is a key C4 SCFA with a wide range of beneficial properties. NaB, which is a salt of butyric acid, may open a new promising option and can be a potential strategy for colorectal cancer patients. It provides anti-tumor effects *via* several molecular mechanisms, and they have been described in this paper. Nevertheless, studies that were published assess the effect of NaB on colorectal cancer cell lines or regard animal model studies. There is a large shortage of studies that investigate the results of supplementation with NaB in patients with colorectal cancer. It can be linked to the popularity of this type of products as well as the formal regulation of NaB, because it may differ depending on country. For instance, in Poland, NaB is available as food with special medical purposes. Overall, NaB should be considered as a supportive product in the complex interdisciplinary anti-cancer treatment.

## Author contributions

All authors read and accepted the current form of the article. All authors contributed to the article and approved the submitted version.

## Conflict of interest

The authors declare that the research was conducted in the absence of any commercial or financial relationships that could be construed as a potential conflict of interest.

## Publisher’s note

All claims expressed in this article are solely those of the authors and do not necessarily represent those of their affiliated organizations, or those of the publisher, the editors and the reviewers. Any product that may be evaluated in this article, or claim that may be made by its manufacturer, is not guaranteed or endorsed by the publisher.
